# Novel Structures for PV Solar Cells: Fabrication of Cu/Cu_2_S-MWCNTs 1D-Hybrid Nanocomposite

**DOI:** 10.3390/mi15111318

**Published:** 2024-10-29

**Authors:** Sevinj Nuriyeva, Aynura Karimova, Habiba Shirinova, Sevinj Jafarova, Ghulam Abbas, Alexandr Zamchiy, Hugo Aguas

**Affiliations:** 1 Nano Research Laboratory, Center of Excellence in Research, Development and Innovation, Baku State University, Baku 1148, Azerbaijan; 2 CENIMAT|i3N, Department of Materials Science, School of Science and Technology, NOVA University Lisbon and CEMOP/UNINOVA, Campus de Caparica, 2829-516 Caparica, Portugal; g.abbas@fct.unl.pt (G.A.);

**Keywords:** copper, copper (I) sulfide, MWCNTs, sulfidation, nanowire, hybrid nanomaterial

## Abstract

The production of cost-effective novel materials for PV solar cells with long-term stability, high energy conversion efficiency, enhanced photon absorption, and easy electron transport has stimulated great interest in the research community over the last decades. In the presented work, Cu/Cu_2_S-MWCNTs nanocomposites were produced and analyzed in the framework of potential applications for PV solar cells. Firstly, the surface of the produced one-dimensional Cu was covered by Cu_2_S nanoflake. XRD data prove the formation of both Cu and Cu_2_S structures. The length and diameter of the one-dimensional Cu wire were 5–15 µm and 80–200 nm, respectively. The thickness of the Cu_2_S nanoflake layer on the surface of the Cu was up to 100 nm. In addition, the Cu/Cu_2_S system was enriched with MWCNTs. MWCNs with a diameter of 50 nm interact by forming a conductive network around the Cu/Cu_2_S system and facilitate quick electron transport. Raman spectra also prove good interfacial coupling between the Cu/Cu_2_S system and MWCNTs, which is crucial for charge separation and electron transfer in PV solar cells. Furthermore, UV studies show that Cu/Cu_2_S-MWCNTs nanocomposites have a wide absorption band. Thus, MWCNTs, Cu, and Cu_2_S exhibit an intense absorption spectrum at 260 nm, 590 nm, and 972 nm, respectively. With a broad absorption band spanning the visible–infrared spectrum, the Cu/Cu_2_S-MWCNTs combination can significantly boost PV solar cells’ power conversion efficiency. Furthermore, UV research demonstrates that the plasmonic character of the material is altered fundamentally when CuS covers the Cu surface. Additionally, MWCN-Cu/Cu2S nanocomposite exhibits hybrid plasmonic phenomena. The bandgap of Cu/Cu_2_S NWs was found to be approximately 1.3 eV. Regarding electron transfer and electromagnetic radiation absorption, the collective oscillations in plasmonic metal-p-type semiconductor–conductor MWCNT contacts can thus greatly increase energy conversion efficiency. The Cu/Cu_2_S-MWCNTs nanocomposite is therefore a promising new material for PV solar cell application.

## 1. Introduction

Sustainable, alternative energy sources are essential as the world’s energy needs grow and environmental issues gain greater attention [1,2,3]. Since solar energy can produce clean, limitless power, it is at the forefront of renewable energy technologies. Nonetheless, the selection of materials for PV solar cell manufacturing has a significant impact on the cells’ efficiency, which is essential to their feasibility. The creation of novel materials or the alteration of preexisting ones to improve their qualities has thus emerged as a key field of study [4,5]. In PV solar cells, more advanced nanostructured materials have replaced conventional amorphous, polycrystalline, and crystalline thin films. Despite their effectiveness, first-generation cells have drawbacks such as expensive production costs, decreased efficiency in low light, and possible environmental effects from certain hazardous constituents [6]. The search for materials that can overcome these limitations has led to exploring nanostructured compounds, which have unique optical and electrical properties that make them suitable for next-generation photovoltaic technologies [7,8,9]. Nanowires (NWs), nanotubes, and nanorods are examples of one-dimensional (1D) nanostructures that have drawn interest because of their high aspect ratio, superior electrical conductivity, and capacity to improve light absorption and scattering [10,11,12,13]. These characteristics make one-dimensional nanostructures the best option for raising PV solar cell efficiency [14,15,16]. Presently, photovoltaics based on zinc sulfide, cadmium telluride, cadmium selenide, copper oxide, titanium oxide, indium-gallium nitride, gallium arsenide, and indium arsenide nanowires are being studied and manufactured [17,18,19]. Among these one-dimensional nanostructures, copper nanowires (Cu NWs) have drawn significant attention because of their high conductivity, low cost, and adjustable optical characteristics, particularly Surface Plasmon Resonance (SPR), which improves light absorption. They are appropriate for energy-related applications such as PV solar cells and lithium-ion batteries (LIBs) due to their strong absorption in the visible light spectrum and good electrical conductivity [20]. One of the main disadvantages of one-dimensional copper is that it is easily oxidized, which lowers its long-term durability in PV solar cells. To enable effective charge percolation through Cu NWs films and networks, the native insulating oxide layer that is present in produced Cu NWs must be eliminated. Numerous attempts have been made to increase copper’s electrochemical stability by coating it with protective shells or creating alloys. Regretfully, these tactics necessitate material post-processing, which raises production costs. Therefore, finding techniques that can protect the Cu surface from oxidation without additional treatment is important. Research has concentrated on integrating Cu NWs with copper sulfide nanostructures to overcome this problem. Copper sulfides, especially Cu_2_S, are well known for their superior optical qualities, chemical stability, affordability, and eco-friendliness. Coating Cu NWs with Cu_2_S increases the material’s optical and electrical qualities additionally to the nanowires’ overall stability. Due to their unique characteristics, which include mixed valence states, non-stoichiometric composition, higher mechanical stability, high electrical conductivity, affordability, nontoxicity, and improved stability under ambient conditions, copper sulfides are a promising electroactive material for advanced energy storage systems. Because of their structural, optical, and electrical characteristics, copper sulfide is particularly regarded as a viable material for solar energy conversion systems as a p-type semiconductor. Cu_2_S structure research has gained major attention lately due to its potential in photovoltaic cells and its numerous technological applications in PV solar cells, tubular solar cells, and photovoltaic solar energy diversions. Research suggests that employing hybrid structures to enhance electron transmission is a better choice for these applications. The amalgamation of several nanostructures to fabricate hybrid materials has emerged as an effective method to enhance solar device performance. A possible technique to improve the efficiency of solar systems is the utilization of hybrid materials which incorporate various nanostructure types.

Cu/Cu_2_S NWs’ incorporation with multi-walled carbon nanotubes (MWCNTs) is one of the most remarkable combinations. MWCNTs are renowned for their notable mechanical flexibility, enormous surface area, and electrical conductivity. MWCNTs can greatly improve charge separation, lower recombination losses, and raise the PV solar cell’s overall efficiency when combined with Cu/Cu_2_S NWs. Cu/Cu_2_S NWs and MWCNTs are two 1D nanostructures that work in cooperation to create a composite material with enhanced physical characteristics. Structures with superior electrical conductivity, mechanical resilience, and high optical transparency can be formed by these hybrid materials [21,22]. They are therefore especially well-suited for usage in transparent, flexible PV solar cells, which are becoming more and more crucial for applications involving next-generation photovoltaics [23].

The intricacy and expense of current synthesis techniques restrict the practical application of Cu/Cu_2_S NWs and MWCNTs-based hybrid materials, despite their encouraging potential. High temperatures, costly chemicals, and intricate procedures that are challenging to scale up for large-scale manufacturing are all part of many existing processes used to create these nanostructures [24]. Simpler, more affordable methods are required to make these materials commercially feasible. Recent developments in synthesis techniques based on solutions present a viable substitute [25,26,27,28,29,30]. Solution-based preparation can be improved to create hybrid materials based on Cu/Cu_2_S NWs and MWCNTs that have the required structural and optical characteristics, opening the door for their wider application in PV solar cell technology in the future.

The goal of this study is to establish a straightforward, scalable method to produce hybrid materials based on Cu/Cu_2_S NWs and MWCNTs. The suggested method aims to solve the scalability and material constraints, advancing the creation of inexpensive, highly effective materials for PV solar cell applications that can satisfy the rising demand for renewable energy. Future investigations into the electrochemical characteristics of these hybrid materials may unveil new opportunities for their utilization in other energy-related domains, including supercapacitors and energy storage systems. By continuing to explore novel materials and improving existing ones, we can pave the way for more efficient and sustainable energy solutions.

## 2. Materials and Methods

### 2.1. Materials

Copper (II) chloride (CuCl_2_, 99%), sodium hydroxide (NaOH, 98%), natrium sulfide (Na_2_S, 99.0%), hydrazine (N_2_H_4_, 35 wt%), and ethylenediamine (C_2_H_8_N_2_, EDA, 99.5%) were purchased from Karma Lab (Izmir, Turkey). MWCNTs (purity: ≥95.0%; 30–50 nm in diameter and 5–20 µm in length) were purchased from Sky Spring Nanomaterials (Houston, TX, USA). All chemicals were of analytical grade and used without further purification.

### 2.2. Synthesis of the Cu and Cu/Cu_2_S NWs

Copper nanowires were synthesized utilizing a solution-based approach with varying chemical concentrations [31]. First, 5 mL of the 9 M NaOH solution was prepared. After 10 min, 50 μL of EDA was added to the NaOH solution. Subsequently, 2 mL of a CuCl_2_ water solution was added to the colorless solution, which was stirred for 10 min. The solution quickly turned bright blue, and, after an additional 10 min, it darkened. To ensure that the reaction proceeded homogeneously, stirring was carried out using a magnetic stirrer with 300 rpm. Furthermore, a water bath was employed to keep the solution’s temperature at a constant 70 °C. The reaction was completed in 2 h. In the first stage of the experiment, Cu(OH)_2_ (copper (II) hydroxide) was formed, and EDA covered its surface, preventing aggregation. Then, 15 μL of N_2_H_4_ solution, diluted with distilled water, was added to the solution as a reducing agent. Initially, the reducing agent reduces Cu^2+^ ions in Cu(OH)_2_ into copper (I) oxide (Cu_2_O) particles. Specifically, this redox reaction occurs as follows:

2Cu(OH)_2_ + N_2_H_4_ → Cu_2_O + N_2_ + 2H_2_O_2_

As a strong reducing agent, N_2_H_4_ donates electrons to Cu^2^⁺ ions, facilitating the conversion. Cu^2^⁺ ions gain electrons to form Cu⁺, while hydrazine loses electrons to form N_2_ gas. Cu_2_O was further reduced to Cu atoms by continuously adding N_2_H_4_ and heating in a water bath, resulting in the formation of Cu NWs—from the generation of seeds to growth. During the 2 h reaction time, the color of the final solution changed from dark blue to reddish brown. Finally, the solution was washed with methanol to remove any remaining chemical contaminants from the produced NWs.

Cu/Cu_2_S NWs were produced by the sulfidation of Cu NWs [32]. In this basic sulfidation technique, sodium sulfide serves as the sulfur source. To prepare Cu/Cu_2_S NWs, 19.5 mg of sodium sulfide (2 mM Na_2_S) was dissolved in 50 mL of ethanol. Cu NWs (1.2 mg/mL) was suspended in 5 mL of ethanol and mixed with the Na_2_S/ethanol solution. To obtain a uniform dispersion, the reaction flask was treated with ultrasonic radiation for 2 min (0.6 cm diameter; Ti-tip; 20 kHz; 30 W/cm^2^) at 300 °C, supported by a cooling bath. Finally, the solution was placed in a vacuum oven adjusted to 80 °C for 2 h without stirring. The Cu/Cu_2_S NWs were then centrifuged several times at 5000 rpm for 10 min to remove the reaction remains from the ethanol. Finally, the synthesized sample was kept in 40 mL of ethanol for future use.

### 2.3. Fabrication of Cu/Cu_2_S NWs-MWCNTs Nanocomposites

Cu/Cu_2_S-MWCNTs nanocomposites were obtained through a simple mixing–drying procedure, which included sonication (20 kHz; 30 W/cm^2^). First, the optimal reagent proportions were determined; the ratio of MWCNTs and Cu/Cu_2_S was chosen as 1:10, respectively. Thus, 0.18 g/L and 1.8 g/L ethanolic solutions of the chemical substances were prepared, subjected to sonication for 2 min, and then mixed for 3 min by adding one to another. The resulting Cu/Cu_2_S-MWCNTs dispersion was centrifuged and dried at 60 °C.

### 2.4. Material Characterization

The ultraviolet–visible (UV–Vis) spectrum was recorded on a spectrophotometer, the Specord250 Plus at a wavelength range between 200 nm and 700 nm. X-ray powder diffraction (XRD) patterns were examined at room temperature using a PANalytical X’pert PRO diffractometer with Cu-Kα radiation. The samples were scanned in the 2θ range of 10–80°. Raman spectra were collected using a Renishaw Qontor equipped with a microscope with a 150 mW 532 nm laser excitation source using a 20× objective. The morphology of the samples was characterized with a Carl Zeiss AURIGA CrossBeam Workstation scanning electron microscope (SEM, Oberkochen, Germany) and a Hitachi HF5000 field-emission scanning transmission electron microscope (STEM, Mito, Ibaraki, Japan) system operated at 200 kV. Materials were processed ultrasonically using the Sonics Vibra-Cell mobil VCX 500 device.

## 3. Results and Discussion

### 3.1. The Morphological and Structural Characterization of Cu and Cu/Cu_2_S NWs, and Cu/Cu_2_S-MWCNTs Nanocomposites

**Figure 1:**
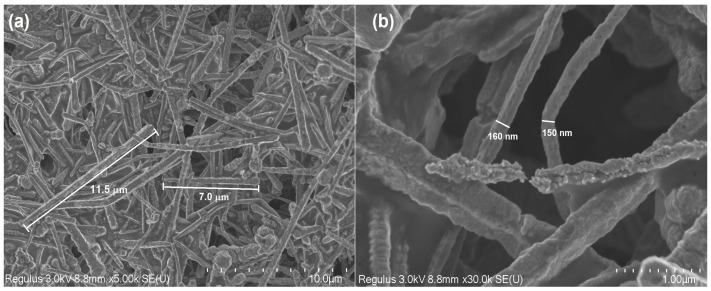
SEM images of Cu NWs at different magnifications: 5 K (**a**); 30 K (**b**).

The morphology and sizes of the produced Cu NWs were examined by the SEM method at various magnifications (Figure 1). SEM images of the Cu NWs reveal that high aspect ratio wires with a cylindrical form were synthesized. The length and diameter of the Cu NWs vary between 5 and 15 µm (Figure 1a), and 80 and 200 nm, respectively (Figure 1b).

**Figure 2:**
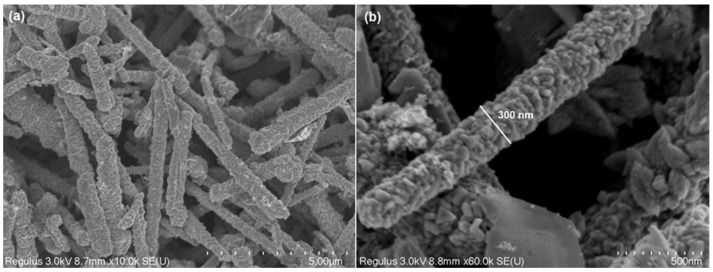
SEM images of Cu/Cu_2_S NWs at different magnifications: 10 K (**a**); 60 K (**b**).

Figure 2 illustrates Cu/Cu_2_S NWs SEM images at various magnifications. The Cu NWs’ surfaces have been coated with Cu_2_S nanoflakes (ultra-thin nanosheets) due to the sulfidation processes. Figure 2a illustrates how the diameter of the initial NWs increased and altered between 150 and 400 nm (Figure 2b). It is known that 1D Cu has much higher electrical conductivity [33]. However, one major drawback of 1D Cu is its susceptibility to oxidation, which reduces its long-term stability in photovoltaic devices. To address this issue, research has focused on combining Cu NWs with copper sulfide (Cu_2_S) nanostructures. Copper sulfides, particularly Cu_2_S, are widely recognized for their excellent optical properties, chemical stability, cost-effectiveness, and environmentally friendly nature [34,35]. In addition, Cu is a good conductor, and its main charge carriers are electrons. Cu_2_S is a p-type semiconductor [36]. When a Cu/Cu_2_S contact is made, due to the different distribution of electrons and holes between the two materials, a Schottky junction is formed. The Schottky junction prevents the free transfer of electrons from metallic Cu to Cu_2_S, which creates the basis for the formation of photovoltaic effects [37,38].

**Figure 3:**
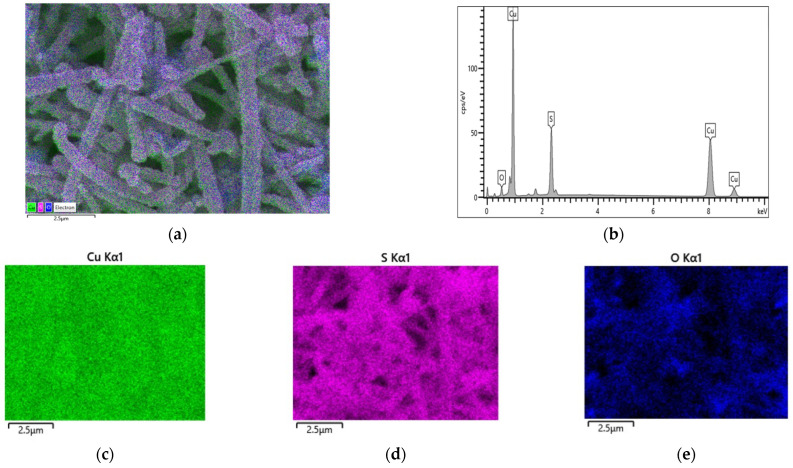
(**a**) SEM–(**b**) EDS analysis of the Cu/Cu_2_S NWs; (**c**) Cu Kα1; (**d**) S Kα1; (**e**) O Kα1.

The elemental analysis and chemical characterization of Cu/Cu_2_S NWs phases were studied using energy-dispersive X-ray microanalysis. The mapping of Cu/Cu_2_S NWs to identify elemental concentrations is shown in Figure 3. Additionally, the results of energy-dispersive X-ray spectra are given in Table 1. Quantitative analysis of the elements in Cu/Cu_2_S NWs has found that the phase is mainly composed of copper, sulfur, and oxygen (Table 1). So, the examined atomic percentage ratio Cu is more than twice, which confirms that the structure formed on Cu NWs is primarily Cu_2_S.

**Figure 4:**
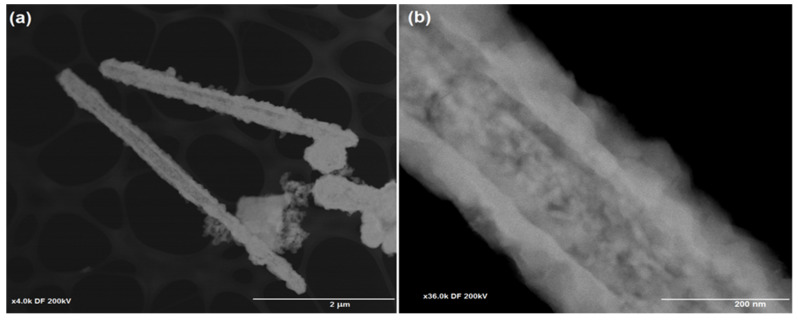
TEM images of Cu/Cu_2_S NWs: 4 K (**a**); 36 K (**b**).

The sizes and morphologies of Cu/Cu_2_S NWs were analyzed through transmission electron microscopy (TEM) (Figure 4). The dimensions of the NWs determined by the TEM study show good agreement with the SEM investigations. The observed structure shows a clear distinction between the Cu and Cu_2_S layers. It is clear from the TEM images that the thickness of the Cu_2_S nanoflake layer on the surface of Cu is up to 100 nm.

**Figure 5:**
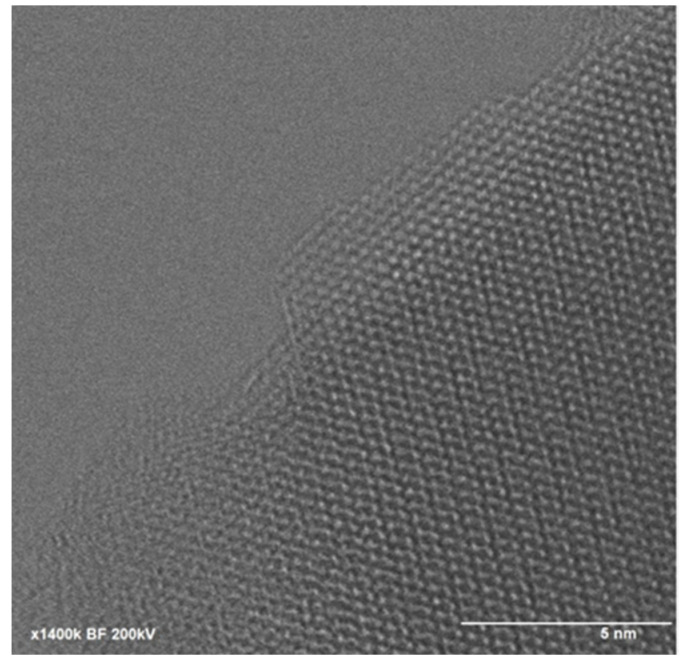
HRTEM image of Cu/Cu_2_S NWs.

Furthermore, high-resolution TEM imaging (Figure 5) can reveal characteristic crystal lattice elements, indicating the presence of crystalline structures with unique morphology. Thus, the structure of Cu/Cu_2_S NWs resembles specific simulations, displaying a crystalline lattice with a hexagonal structure (Figure 5).

**Figure 6:**
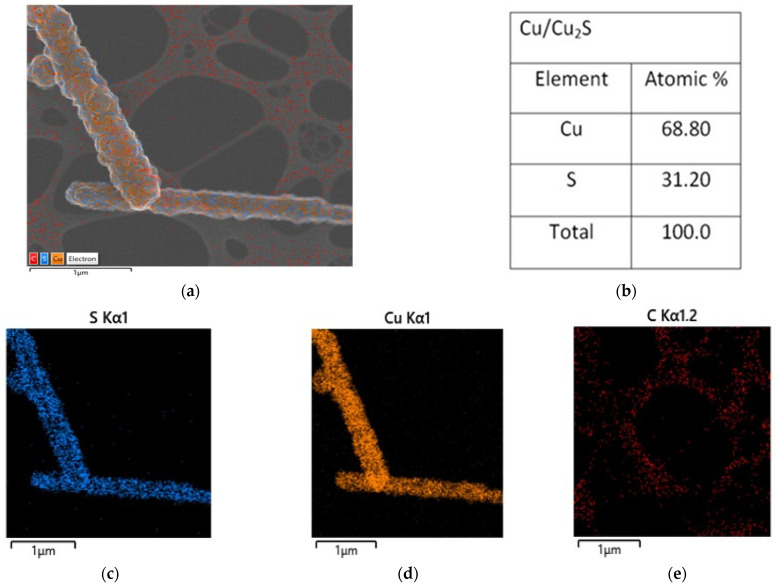
(**a**) TEM EDS analysis of the Cu/Cu_2_S NWs; (**b**) atomic percentage; (**c**) S Kα1; (**d**) Cu Kα1; (**e**) C Kα1.

The overlapping of copper and sulfur elements in the mapping image from TEM shows that the formed structures were Cu/Cu_2_S NWs (Figure 6). The atomic percentage of the elements was 68.80 atomic % and 31.20 atomic % in copper and sulfur, respectively, which is consistent with the SEM data and suggests that the Cu:S ratio was 2.2.

**Figure 7:**
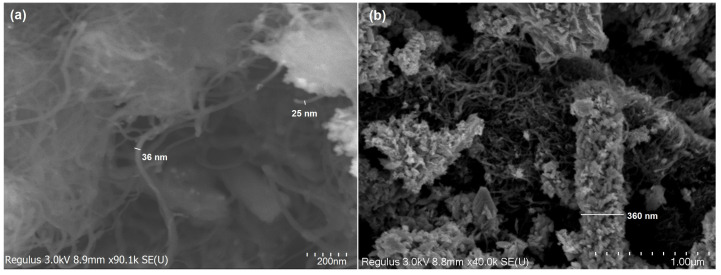
SEM images of MWCNTs (**a**) and Cu/Cu_2_S-MWCNTs (**b**) nanocomposites.

In this investigation, MWCNTs were also added to the Cu/Cu_2_S system to enhance the electron transport of electrons. The transport of electrons across the network of nanometer-sized particles inside the material is recognized to be a significant obstacle to achieving improved energy conversion efficiency [39,40,41]. The morphology of Cu/Cu_2_S-MWCNTs nanocomposites was examined through the SEM method at different magnifications (Figure 7). SEM images confirm the strong connection between the Cu/Cu_2_S NWs and MWCNTs. Cu/Cu_2_S wires are interlinked closely with MWCNTs. MWCNTs create a network around Cu/Cu_2_S NWs. Moreover, the interaction between MWCNTs and Cu/Cu_2_S could facilitate quick electronic transport by forming interlinked conducting networks [42,43]. Such tight and immediate connections (contacts) between Cu/Cu_2_S and MWNTs can provide a direct route for charge transfer and lead to considering the nanocomposite as an appropriate electrode material [43]. It is significant to state that the direct electrostatic contact between Cu/Cu_2_S NWs and MWCNTs may generate interfacial coupling and separation that is effective for charge carrier transfer [44].

### 3.2. Structural and Optical Characteristics of Cu, Cu/Cu_2_S NWs and Cu/Cu_2_S-MWCNTs Nanocomposites

**Figure 8:**
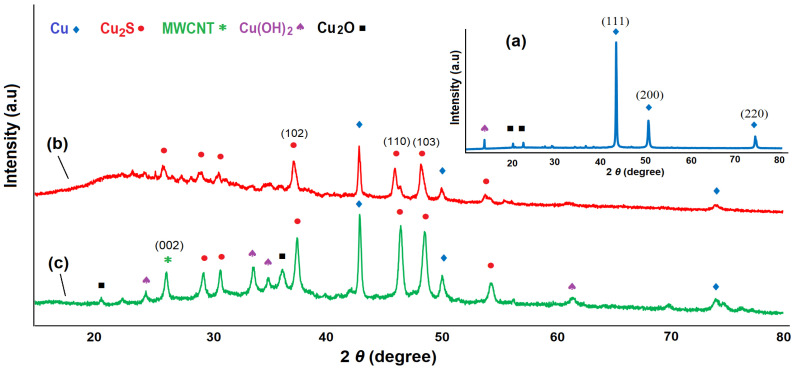
X-ray diffraction patterns of Cu NWs (a), Cu/Cu_2_S NWs (b), and Cu/Cu_2_S-MWCNTs (c) nanocomposites.

The X-ray diffractograms of Cu NWs, Cu/Cu_2_S NWs, and Cu/Cu_2_S-MWCNTs nanocomposites are given in Figure 8. The peaks of Cu at 2θ = 43.26°, 50.41°, and 74.02° correspond to planes (111), (200), and (220), respectively, indicating a face-centered cubic lattice [45,46,47]. In addition, Cu(OH)_2_ and Cu_2_O XRD patterns also appeared at 2θ = 14.04°, 20.31°, and 22.72° [48]. The presence of oxygen can be explained by the fact that complete sulfidation of copper NTs does not occur, but the dicopper oxide phase remains.

After sulfidation of Cu NWs, the XRD pattern demonstrates peaks at 26.28°, 29.69°, 31.12°, 37.59°, 43.29°, 46.41°, 48.64°, 50.42°, 54.20°, and 74.10°. The peaks at 2θ = 26.28°, 29.69°, 31.12°, 37.59°, 46.41°, 48.64°, and 54.20° correspond to the hexagonal phase of copper (I) sulfide, specifically the chalcocite phase Cu_2_S [49]. A pattern expansion between 10 and 30° in XRD is also seen, indicating the creation of an amorphous phase. Amorphous phase presence suggests that the structure formed on the surface of Cu NWs is Cu_2_S [50,51]. Notably, the structure generated in the coating layer throughout the sulfidation procedure could include various mixed phases of copper sulfide (CuS, Cu_9_S_5_, Cu_2_S, and others), and the formed copper sulfide layer might protect copper against oxidation. The HRTEM and XRD analysis results indicate the structure on the Cu NWs is most probably a p-type semiconductor Cu_2_S with a hexagonal phase.

Figure 8c depicts the XRD pattern of the Cu/Cu_2_S-MWCNTs nanocomposite. Diffraction at 26.53° corresponds to the (002) plane of the carbon nanotubes’ graphite layer with hexagonal lattice [52]. The diffraction peaks corresponding to Cu/Cu_2_S NWs remain nearly the same after the MWCNTs’ addition, indicating that the phase structure has not changed considerably. The XRD pattern shows good agreement with the SEM data, indicating strong linking between Cu/Cu_2_S NWs and MWCNTs [53].

**Figure 9:**
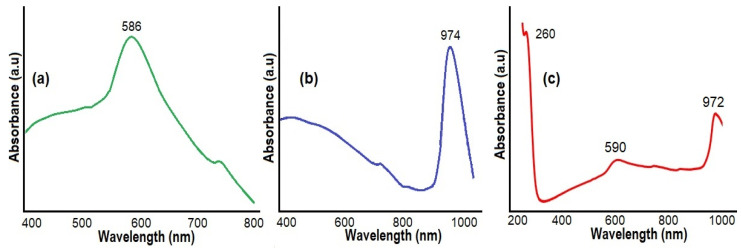
UV–Vis absorption spectrum of Cu NWs (**a**), Cu/Cu_2_S NWs (**b**), and Cu/Cu_2_S-MWCNTs nanocomposites (**c**).

It is known that the material’s surface plasmon resonance (SPR) feature also plays a crucial role in PV solar cells’ efficiency [54,55]. The SPR effect helps to collect light energy more effectively and increases the PV solar cell’s efficiency [56,57,58,59]. The absorption spectra of Cu, Cu/Cu_2_S NWs, and Cu/Cu_2_S-MWCNTs nanocomposites were investigated to study the (SPR) feature (Figure 9). The significant peak seen at 586 nm is due to the surface plasmon absorption of Cu nanostructures. Its short-wavelength tail is associated with d-sp interband transitions (Figure 9a).

The UV–Vis absorption spectrum of Cu/Cu_2_S NWs (Figure 9b) shows a fundamental change in the plasmonic nature of the material and hybrid plasmonic effects. The absorption peak of Cu wires broadens at 586 nm. The absorption band broadness can be related to the interaction of the Cu NWs with Cu_2_S flake layers. Such collective oscillations occurring in metal-p-type semiconductor contacts can substantially increase energy conversion efficiency in terms of absorption of electromagnetic rays and electron transitions [57]. The strong near-infrared absorption is generated by the collective oscillation of free charge carriers (holes) in the valence band on the surface of Cu/Cu_2_S NWs, resulting in the LSPR effect [58]. A maximum at 974 nm in the near-infrared region was observed, which is characteristic of Cu_2_S and related to the local surface plasmon resonance (LSPR) phenomenon. The LSPR peaks are at wavelengths ranging from 900 to 1400 nm, depending on the structure’s form and composition [59,60].

Figure 9c displays the UV–Vis absorption spectrum of the Cu/Cu_2_S-MWCNTs nanocomposite. The Cu/Cu_2_S-MWCNTs hybrid structures exhibit the characteristic absorption peaks of pristine MWCNTs at 260 nm, which correspond to absorption bands of phenyl groups [61]. The size effect of nanowires and nanotubes causes shifts in the Cu and Cu-Cu_2_S NW peaks at 590 and 972 nm, respectively [58,59]. With all that said, UV studies show that Cu/Cu_2_S-MWCNTs have a broad absorption band. Consequently, the Cu/Cu_2_S-MWCNTs system, having a broad absorption band covering the visible–infrared range, can lead to a substantial increase in the power conversion efficiency in PV solar cells.

**Figure 10:**
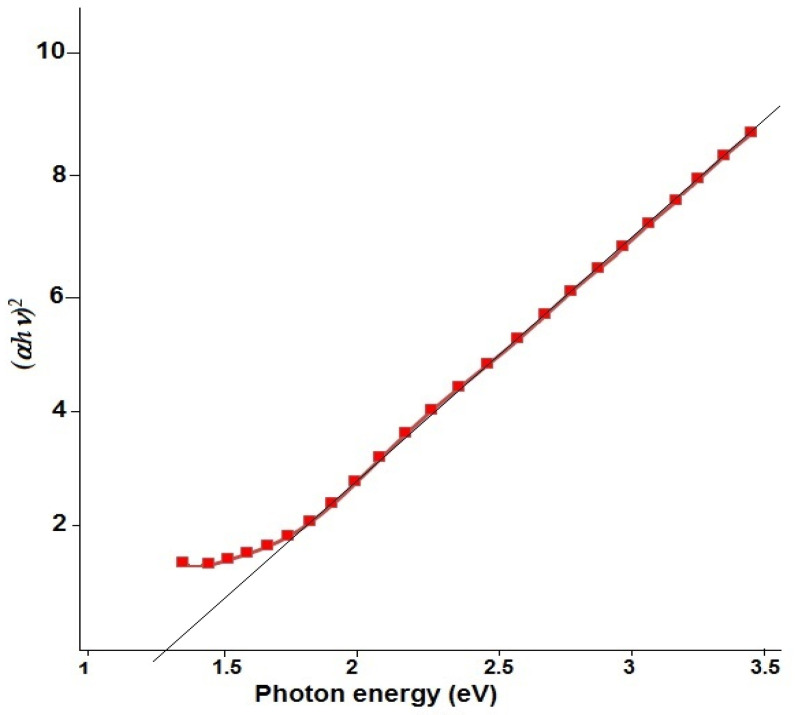
The bandgap energy of Cu/Cu_2_S NWs’.

Figure 10 depicts the bandgap calculation for Cu/Cu_2_S nanostructures. The bandgap of Cu/Cu_2_S NWs was determined using the Tauc plot [62] and found to be approximately 1.3 eV, with a blue shift due to the particles’ smaller size, compared to the bulk phase of Cu_2_S (1.2 eV) [63]. The investigation of the bandgap value also revealed that the structures observed on the surface of Cu NWs were Cu_2_S. It can be concluded that the structure formed at values less than 1.5 eV is Cu_2_S [64,65,66].

**Figure 11:**
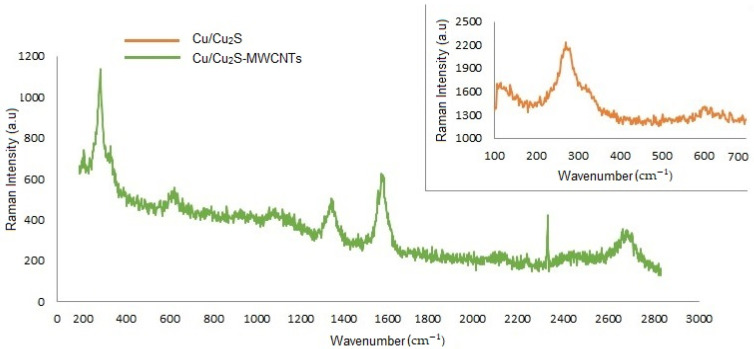
Raman spectrum of Cu/Cu_2_S NWs and Cu/Cu_2_S-MWCNTs nanocomposites.

Raman spectroscopy was implemented to investigate the structural modifications of the Cu/Cu_2_S NWs and Cu/Cu_2_S-MWCNTs nanocomposite (Figure 11).

Cu/Cu_2_S NWs exhibit a noticeable Raman band in the 270–280 cm^−1^ range, attributed to vibrational oscillations of the Cu–S phase and copper oxide structures. The weak signal at 620 cm^−1^ could be attributed to vibrations of oxide groups [67,68,69]. Raman spectroscopy of Cu/Cu_2_S-MWCNTs nanocomposites shows sharp peaks at 280, 329, 629, 1350, 1583, 2329, and 2700 cm^−^^1^. The Raman maxima at 1350, 1583, and 2700 represent the D band (defect), G band (graphite band), and G′ band (D tone) of MWCNTs, respectively. The shift of the Raman maxima at 1350, 1583, and 2700 cm^−1^ to a higher wavenumber compared to that of pure MWCNTs occurs due to structural defects of nanocomposites, which cause charge transfer between the components through modification [70]. Furthermore, this shift indicates that structural interactions are occurring between Cu/Cu_2_S and MWCNTs, which is also a crucial factor for electron transfer.

## 4. Conclusions

Over the past few decades, the research community has become very interested in novel materials development for photovoltaic solar cells that possess high energy conversion efficiency, improved photon absorption, long-term stability, and easy electron transport. In this study, Cu/Cu_2_S-MWCNTs nanocomposites are synthesized successfully, demonstrating the material’s encouraging potential for use in next-generation photovoltaic solar cell applications. The experimental findings validate the development of a stable Cu/Cu_2_S structure in which a protective layer of Cu_2_S nanoflakes covers the Cu wires. The thickness of the Cu_2_S nanoflake layer on the surface of Cu wires is up to 100 nm. The incorporation of MWCNTs with a 50 nm diameter further enhances the material by forming a conductive network that facilitates charge separation at the interfaces and highly increases electron transport. Raman and UV–Vis spectra also reveal that this interconnected network of Cu NWs, Cu_2_S-nanoflake layer, and MWCNTs offers strong interfacial coupling and facilitates effective charge carrier transfer. Furthermore, the hybrid plasmonic effects are observed in the composite material. Particularly, the broad absorption spectrum covering visible to near-infrared ranges highlights the Cu/Cu_2_S-MWCNTs nanocomposite’s capacity for enhanced photon absorption. Thus, MWCNTs, Cu, and Cu_2_S exhibit an intense absorption spectrum at 250 nm, 583 nm, and 974 nm, respectively. This broad absorption spectrum, combined with the localized surface plasmon resonance (LSPR) characteristics, makes the Cu/Cu_2_S-MWCNTs nanocomposite highly suitable for improving the PV solar cells’ power conversion efficiency.

It is important to emphasize that this research focuses on the material level, providing an essential basis for future exploration into device fabrication rather than electrode development. The synthesized nanocomposite represents a foundational material for PV solar cells and demonstrates its potential in addressing challenges such as stability, light absorption, and charge transport. Since Cu/Cu_2_S-MWCNTs nanocomposite possesses enhanced charge carrier transfer due to the metal Cu, p-type semiconductor Cu_2_S, and conductive MWCNT combination, this material can play the role electron transport layer to improve electron mobility and collection efficiency of PV solar cells.

## Figures and Tables

**Table 1:** The results of energy dispersive X-ray spectra of Cu/Cu_2_S NWs.

Element	Line Type	Apparent Concentration	K Ratio	wt%	at%
Cu	K series	31.35	0.31354	76.83	56.32%
S	K series	4.58	0.03943	16.55	24.26%
O	K series	1.27	0.00428	6.62	19.42%
Total	100.0				100.0

## Data Availability

The original contributions presented in this study are included in the article. Further inquiries can be directed to the corresponding author(s).

## References

[1] Aricò A.S., Bruce P., Scrosati B., Tarascon J., Van Schalkwijk W. (2005). Nanostructured materials for advanced energy conversion and storage devices. Nat. Mater..

[2] Liu C., Li F., Ma L., Cheng H. (2010). Advanced materials for energy storage. Adv. Mater..

[3] Yoo H.D., Markevich E., Salitra G., Sharon D., Aurbach D. (2014). On the challenge of developing advanced technologies for electrochemical energy storage and conversion. Mater. Today.

[4] Gao M., Xu Y., Jiang J., Yu S. (2013). CHEmInFORM Abstract: Nanostructured Metal chalcogenides: Synthesis, modification, and applications in energy conversion and storage devices. ChemInform.

[5] Balaya P. (2008). Size effects and nanostructured materials for energy applications. Energy Environ. Sci..

[6] Iqbal M.A., Malik M., Shahid W., Din S.Z.U., Anwar N., Ikram M., Idrees F. (2022). Materials for Photovoltaics: Overview, generations, recent advancements and future prospects. IntechOpen eBooks.

[7] Kislyuk V.V., Dimitriev O.P. (2008). Nanorods and nanotubes for solar cells. J. Nanosci. Nanotechnol..

[8] Bist A., Pant B., Ojha G.P., Acharya J., Park M., Saud P.S. (2023). Novel materials in perovskite solar cells: Efficiency, stability, and future perspectives. Nanomaterials.

[9] Tawalbeh M., Al-Othman A., Kafiah F., Abdelsalam E., Almomani F., Alkasrawi M. (2021). Environmental impacts of solar photovoltaic systems: A critical review of recent progress and future outlook. Sci. Total Environ..

[10] Hochbaum A.I., Yang P. (2009). Semiconductor nanowires for energy conversion. Chem. Rev..

[11] Abd-Ellah M., Thomas J.P., Zhang L., Leung K.T. (2016). Enhancement of solar cell performance of p-Cu_2_O/n-ZnO-nanotube and nanorod heterojunction devices. Sol. Energy Mater. Sol. Cells.

[12] Tang J., Huo Z., Brittman S., Gao H., Yang P. (2011). Solution-processed core–shell nanowires for efficient photovoltaic cells. Nat. Nanotechnol..

[13] Czaban J.A., Thompson D.A., LaPierre R.R. (2009). GAAs Core−Shell nanowires for photovoltaic applications. Nano Lett..

[14] Tian B., Zheng X., Kempa T.J., Fang Y., Yu N., Yu G., Lieber C.M. (2007). Coaxial silicon nanowires as solar cells and nanoelectronic power sources. Nature.

[15] Cao Y., Bernechea M., Maclachlan A., Zardetto V., Creatore M., Haque S.A., Konstantatos G. (2015). Solution Processed bismuth sulfide nanowire array Core/Silver sulfide shell solar cells. Chem. Mater..

[16] Garnett E.C., Yang P. (2008). Silicon Nanowire Radial p−n Junction Solar Cells. J. Am. Chem. Soc..

[17] Law M., Greene L.E., Johnson J.C., Saykally R., Yang P. (2005). Nanowire dye-sensitized solar cells. Nat. Mater..

[18] Wang K., Chen J.J., Zeng Z.M., Tarr J., Zhou W.L., Zhang Y., Mascarenhas A. (2010). Synthesis and photovoltaic effect of vertically aligned ZnO/ZnS core/shell nanowire arrays. Appl. Phys. Lett..

[19] Greene L.E., Law M., Yuhas B.D., Yang P. (2007). ZnO−TiO_2_ Core−Shell NanoROD/P3HT Solar Cells. J. Phys. Chem. C.

[20] Cheng L., Zhong Y., Wang Q., Zhou L. (2022). In Situ Partial Sulfidation for Preparing Cu/Cu_2−x_S Core/Shell Nanorods with Enhanced Photocatalytic Degradation. Catalysts.

[21] Lee J., Connor S.T., Cui Y., Peumans P. (2008). Solution-Processed Metal nanowire mesh transparent electrodes. Nano Lett..

[22] Rowell M.W., Topinka M.A., McGehee M.D., Prall H., Dennler G., Sariciftci N.S., Gruner G. (2006). Organic solar cells with carbon nanotube network electrodes. Appl. Phys. Lett..

[23] Chen P., Shen G., Shi Y., Chen H., Zhou C. (2010). Preparation and characterization of flexible asymmetric supercapacitors based on Transition-Metal-Oxide Nanowire/Single-Walled carbon nanotube hybrid Thin-Film electrodes. ACS Nano.

[24] Jaldurgam F.F., Ahmad Z., Touati F. (2021). Synthesis and performance of Large-Scale Cost-Effective Environment-Friendly nanostructured thermoelectric materials. Nanomaterials.

[25] Han J.H., Kwak M., Kim Y., Cheon J. (2018). Recent advances in the Solution-Based preparation of Two-Dimensional Layered Transition metal chalcogenide nanostructures. Chem. Rev..

[26] Shirinova H., Surkhayli A., Pashayev B., Mammadov H., Jafarov M., Gahramanli L. (2024). Preparation, characterization and thermal properties of the PS+Si based polymer nanocomposites. J. Thermoplast. Compos. Mater..

[27] Shirinova H.A. (2024). Electron spin Resonance Study of magnetite Nanoparticles in IPP Matrix. J. Miner. Mater. Sci..

[28] Nuriyeva S., Shirinova H., Hasanov K., Hajiyeva F. (2023). Controlled Synthesis of Silver nanowires: Production and Characterization. Acta Phys. Pol. A.

[29] Ramazanov M., Hajiyeva F., Babayev Y., Valadova G., Nuriyeva S., Shirinova H. (2019). Synthesis and optical properties of PVC-CdS-based nanocomposites. J. Elastomers Plast..

[30] Zhang X., Pollitt S., Jung G., Niu W., Adams P., Bühler J., Tilley S.D. (2023). Solution-Processed Cu_2_S nanostructures for solar hydrogen production. Chem. Mater..

[31] Lee S. (2000). The Optimized Synthesis of Copper Nanowire for High-Quality and Fabrication of Core-Shell Nanowire. Master’s Thesis.

[32] Anichini C., Czepa W., Aliprandi A., Consolaro V.G., Ersen I., Ciesielski A., Samorì P. (2021). Synthesis and characterization of ultralong copper sulfide nanowires and their electrical properties. J. Mater. Chem. C.

[33] Ma L., Zhang J., Xu K. (2012). Structural and electronic properties of ultrathin copper nanowires: A density-functional theory study. Phys. B Condens. Matter.

[34] Jung J., Jeon H.J., Yang S.W., Choi M., Vidyasagar D., Kim J.H., Shim R.B., Yun Y., Han S., Cho I.S. (2023). Cost-effective synthesis of copper sulfide nanoparticles and flexible films for photocatalytic and antibiotic applications. J. Mater. Res. Technol..

[35] Kozhevnikova N.S., Maskaeva L.N., Markov V.P., Lipina O.A., Chufarov A.U., Kuznetsov M.V. (2019). One-pot green synthesis of copper sulfide (I) thin films with p-type conductivity. Mater. Chem. Phys..

[36] Jiang Y., Xu Y., Zhang Q., Zhao X., Xiao F., Wang X., Ma G. (2024). Templated synthesis of Cu_2_S hollow structures for highly active ozone decomposition. Catalysts.

[37] Qiu P., Zhang Y., Cheng G. (2022). Precursor self-derived Cu@TiO_2_ hybrid Schottky junction for enhanced solar-to-hydrogen evolution. Int. J. Hydrogen Energy.

[38] Naik S.G., Rabinal M.K., Datta S. (2023). Soft grafting of DNA over hexagonal copper sulfide for low-power memristor switching. Mater. Adv..

[39] Ye Z., Wang N., Gao Y., Cheng Y., Zan L., Fu F., Wei Q. (2023). High photoelectric conversion efficiency and stability of carbon-based perovskite solar cells based on sandwich-structured electronic layers. Colloids Surf. A Physicochem. Eng. Asp..

[40] Fella C.M., Uhl A.R., Hammond C., Hermans I., Romanyuk Y.E., Tiwari A.N. (2013). Formation mechanism of Cu_2_ZnSnSe_4_ absorber layers during selenization of solution deposited metal precursors. J. Alloys Compd..

[41] Aftab S., Abbas A., Iqbal M.Z., Hussain S., Kabir F., Hegazy H.H., Xu F., Kim J.H., Goud B.S. (2023). Two-dimensional MXene incorporating for electron and hole transport in high-performance perovskite solar cells. Mater. Today Energy.

[42] Zhang L., Gong H. (2015). Partial Conversion of Current Collectors into Nickel Copper Oxide Electrode Materials for High-Performance Energy Storage Devices. ACS Appl. Mater. Interfaces.

[43] Rani L., Han J.I. (2024). Fabrication of CuS/Cu_2_S nanoparticles integrated with multi-walled carbon nanotubes for advanced energy storage applications. J. Energy Storage.

[44] Jathar S.B., Rondiya S.R., Jadhav Y.A., Nilegave D.S., Cross R.W., Barma S.V., Nasane M.P., Gaware S.A., Bade B.R., Jadkar S.R. (2021). Ternary Cu_2_SNS_3_: Synthesis, structure, photoelectrochemical activity, and heterojunction band offset and alignment. Chem. Mater..

[45] Lewis C.S., Wang L., Liu H., Han J., Wong S.S. (2014). Synthesis, Characterization, and Formation Mechanism of Crystalline Cu and Ni Metallic Nanowires under Ambient, Seedless, Surfactantless Conditions. Cryst. Growth Des..

[46] Liu Y., Zhang M., Wang F., Pan G. (2012). Facile microwave-assisted synthesis of uniform single-crystal copper nanowires with excellent electrical conductivity. RSC Adv..

[47] Guo H., Lin N., Chen Y., Wang Z., Xie Q., Zheng T., Peng D. (2013). Copper nanowires as fully transparent conductive electrodes. Sci. Rep..

[48] Wang L., Zhang K., Hu Z., Duan W., Cheng F., Chen J. (2013). Porous CuO nanowires as the anode of rechargeable Na-ion batteries. Nano Res..

[49] Liu Z., Xu D., Liang J., Shen J., Zhang S., Qian Y. (2005). Growth of Cu_2_S ultrathin nanowires in a binary surfactant solvent. J. Phys. Chem. B.

[50] Lu S., Yin L., Xin C., Yang X., Chi M., Wan W., Han Y., Zhang L., Zhang P. (2023). Resin-derived carbon to in-situ support Cu-Cu_2−x_S heteroparticles for efficient photocatalytic reduction of Cr(VI). Mol. Catal..

[51] Ma R., Stegemeier J., Levard C., Dale J.G., Noack C.W., Yang T., Lowry G.V. (2014). Sulfidation of copper oxide nanoparticles and properties of resulting copper sulfide. Environ. Sci. Nano.

[52] Saravanan A., Prasad K., Gokulakrishnan N., Kalaivani R., Somanathan T. (2014). Efficiency of transition metals in combustion catalyst for high yield helical Multi-Walled carbon nanotubes. Adv. Sci. Eng. Med..

[53] Zhan Z., Liu C., Zheng L., Sun G., Li B., Zhang Q. (2011). Photoresponse of multi-walled carbon nanotube–copper sulfide (MWNT–CuS) hybrid nanostructures. Phys. Chem. Chem. Phys..

[54] Ali M., Zhou F., Chen K., Kotzur C., Xiao C., Bourgeois L., Zhang X., MacFarlane D.R. (2016). Nanostructured photoelectrochemical solar cell for nitrogen reduction using plasmon-enhanced black silicon. Nat. Commun..

[55] Deb S.K. (2007). Opportunities and challenges in science and technology of WO_3_ for electrochromic and related applications. Sol. Energy Mater. Sol. Cells.

[56] Tooghi A., Fathi D., Eskandari M. (2020). High-performance perovskite solar cell using photonic–plasmonic nanostructure. Sci. Rep..

[57] Krishna MV R., Friesner R.A. (1991). Quantum confinement effects in semiconductor clusters. J. Chem. Phys..

[58] Boriskina S.V., Ghasemi H., Chen G. (2013). Plasmonic materials for energy: From physics to applications. Mater. Today.

[59] Gellini C., Ricci M., Feis A. (2023). Copper sulfide small nanoparticles as efficient contrast agent for photoacoustic imaging. Photonics.

[60] Xu Z., Rao N., Tang C., Cheng C., Law W. (2019). Phase synthesis of Cu_2−x_S nanostructures and their photothermal generation study. ACS Omega Aqueous.

[61] Kaynak N., Önen A., Karahasanoğlu M. (2018). Photoactive multi-walled carbon nanotubes: Synthesis and utilization of benzoin functional MWCNTs. J. Mater. Sci..

[62] Makuła P., Pacia M., Macyk W. (2018). How to correctly determine the band gap energy of modified semiconductor photocatalysts based on UV–VIS spectra. J. Phys. Chem. Lett..

[63] Yu X., An X. (2010). Controllable hydrothermal synthesis of Cu_2_S nanowires on the copper substrate. Mater. Lett..

[64] He L., Zhou D., Lin Y., Ge R., Hou X., Sun X., Zheng C. (2018). Ultrarapid in Situ Synthesis of Cu_2_S Nanosheet Arrays on Copper Foam with Room-Temperature-Active Iodine Plasma for Efficient and Cost-Effective Oxygen Evolution. ACS Catal..

[65] Wu C., Pan Z., Liu Z., Wang Y., Liang F., Yu Y., Luo L. (2017). Controllable synthesis of p-type Cu_2_S nanowires for self-driven NIR photodetector application. J. Nanoparticle Res..

[66] Van Oversteeg CH M., Oropeza F.E., Hofmann J.P., Hensen E.J.M., De Jongh P.E., De Mello Donega C. (2018). Water-Dispersible Copper sulfide nanocrystals via ligand exchange of 1-Dodecanethiol. Chem. Mater..

[67] Liu J., Xue D. (2010). Rapid and scalable route to CuS biosensors: A microwave-assisted Cu-complex transformation into CuS nanotubes for ultrasensitive nonenzymatic glucose sensor. J. Mater. Chem..

[68] Palve B.M., Kadam V.S., Jagtap C.V., Jadkar S.R., Pathan H.M. (2017). A simple chemical route to synthesis the CuSe and CuS counter electrodes for titanium oxide based quantum dot solar cells. J. Mater. Sci. Mater. Electron..

[69] Kahsay A.W., Ibrahim K.B., Tsai M., Birhanu M.K., Chala S.A., Su W., Hwang B. (2019). Selective and low overpotential electrochemical CO_2_ reduction to formate on CuS decorated CuO heterostructure. Catal. Lett..

[70] Mohan S., Oluwafemi O., Songca S., Rouxel D., Miska P., Lewu F., Thomas S. (2016). Completely green synthesis of silver nanoparticle decorated MWCNT and its antibacterial and catalytic properties. Pure Appl. Chem..

